# Accuracy of Machine Learning in Predicting Post‐Stroke Depression: A Systematic Review and Meta‐Analysis

**DOI:** 10.1002/brb3.70557

**Published:** 2025-05-26

**Authors:** Husile Husile, Qinglin Bao, Sarula Sarula, Chu La, Wujisiguleng Wujisiguleng, Siqintu Siqintu, Temuqile Temuqile

**Affiliations:** ^1^ Inner Mongolia Medical University Hohhot Inner Mongolia China; ^2^ International Mongolian Hospital of Inner Mongolia Hohhot Inner Mongolia China; ^3^ College of Traditional Chinese Medicine Beijing University of Chinese Medicine Beijing China

**Keywords:** post‐stroke depression, prediction model, systematic review

## Abstract

**Introduction:**

Post‐stroke depression is one of the important complications of stroke and affects patients' quality of life. Early identification of post‐stroke depression is crucial for its timely prevention. The accuracy of machine learning as a prediction method is controversial. To systematically analyze these studies, we conducted a systematic evaluation to review the effectiveness of the machine learning prediction models in predicting post‐stroke depression based on meta‐analysis.

**Methods:**

As of November 20, 2023, we conducted a systematic literature retrieval in databases such as PubMed, Embase, Cochrane, and Web of Science to investigate machine learning model predictions in patients who had post‐stroke depression. The tool used in this study to assess the quality of the retrieved literature is PROBAST (Prediction model Risk of Bias Assessment Tool).

**Results:**

A total of 28 studies with 85,223 patients as subjects were included in the meta‐analysis. According to data, the c‐index in the validation set was significantly lower than that in the training set and may have been at risk of overfitting, even though there was a desirable accuracy. In addition, we found substantial differences in the duration of follow‐up for post‐stroke depression, and meta‐regression showed that the c‐index based on the prediction models did not decay with longer follow‐up.

**Conclusions:**

Reasonable prediction models are effective prediction tools for post‐stroke depression. A reasonable prediction seems to predict the risk of post‐stroke depression occurrence at different time points and can provide a prevention tool specific to the risk.

## Graphical abstract

• A reasonable prediction seems to predict the risk of post‐stroke depression occurrence at different time points and can provide a prevention tool specific to the risk.• Reasonable prediction models are effective prediction tools for post‐stroke depression.

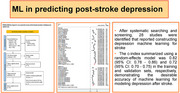

## 1 Introduction

Stroke ranks as the second leading cause of death worldwide, posing a significant threat to public health (Collaborators 2021). Among all stroke occurrences, 87% are ischemic, 10% are due to intracranial hemorrhage, and 3% are attributed to subarachnoid hemorrhage (Saini et al. 2021). Hemorrhagic stroke is associated with higher rates of readmission and mortality compared to ischemic stroke, particularly within the first month following the event (Sloane et al. 2024). While strokes diminish the quality of life for older adults, there has been a noticeable increase in stroke incidence among younger individuals in recent years, with young strokes representing 10–15% of all stroke cases according to statistics (Potter et al. 2022).

Regardless of post‐acute stroke or post‐secondary prevention intravenous thrombolysis or thrombectomy (Mead et al. 2023; Appelros and Terént 2015), some serious psychiatric complications frequently arise (such as post‐stroke cognitive impairment, post‐stroke depression, etc.) (Lekoubou et al. 2023). Post‐stroke depression, or PSD, often develops after a stroke and may significantly impact patients’ functional outcomes (Zhu et al. 2022). It also adds to the challenges faced by stroke survivors and their families. According to statistics, the pooled prevalence of PSD is 27% (95% CI: 25 ‐ 30) (Liu et al. 2023), and it is a risk factor for post‐stroke all‐cause mortality (Cai et al. 2019; Chun et al. 2022). Therefore, early risk prediction for PSD and the development of validated prognostic tools hold critical clinical significance.

Recent developments in machine learning (ML) and deep learning—subfields of artificial intelligence specialized in pattern recognition—have shown great promise in enhancing stroke diagnosis and predicting outcomes (Bonkhoff and Grefkes 2022). ML‐driven predictive analytics improve clinical decision‐making through automated processing of complex datasets, thereby facilitating precision in disease prevention, diagnosis, and ongoing monitoring (Bonkhoff and Grefkes 2022; Li et al. 2023; Deo 2015). Although post‐stroke depression is widely accepted by researchers, effective early prediction methods remain lacking. The researchers have focused primarily on risk factor analysis, which does not accurately identify patients at high risk for post‐stroke complications. ML techniques, however, appear to offer a more accurate means of identifying patients at elevated risk for PSD. Consequently, some researchers have also attempted to develop ML models for predicting post‐stroke depression. Despite these advancements, the predictive capabilities of ML models are not yet fully established. Therefore, a meta‐analysis of ML for the early prediction of PSD has been conducted to advance the development and update of simple tools in this area and to strengthen evidence‐based practices.

## 2 Methods

### 2.1 Study Registration

This study was a meta‐analysis‐based systematic evaluation, which was conducted in accordance with the Preferred Reporting Items for Systematic Reviews and Meta‐Analyses 2020 (i.e., PRISMA 2020) and was prospectively registered in PROSPERO (registration number: CRD42024497464).

### 2.2 Eligibility Criteria

Inclusion Criteria:

(1) Studies that included stroke patients as subjects;
(2) Original studies such as case‐control, nested case‐control, cohort, and case‐cohort studies;
(3) Studies that developed risk prediction models for post‐stroke depression;
(4) Some studies that only performed cross‐validation without external validation, considering that the contribution of these studies should not be overlooked;
(5) A limited number of original studies utilizing different machine learning models on the same data set;
(6) Literature published in English.

Exclusion Criteria:

(1) Studies based on meta‐analyses, reviews, guidelines, expert opinions, or other similar types;
(2) Studies that conducted risk factor analysis without machine learning modeling;
(3) Studies without some outcome measures (ROC, c‐statistic, c‐index, sensitivity, specificity, accuracy, recall, precision, confusion matrix, diagnostic fourfold table, F1 score, calibration curve, etc.) for assessing the accuracy of prediction by machine learning models;
(4) Studies focused exclusively on validating established scales.

## 3 Data Sources and Search Strategy

Utilizing MeSH, we retrieved studies in databases such as Cochrane, Embase, PubMed, and Web of Science from the establishment date of each database, respectively, to December 20, 2023, systematically. There were no restrictions for entry terms on languages or regions. Refer to Supplementary **Table** **S1** for details.

### 3.1 Screening of Studies and Data Extraction

All articles that were retrieved from the abovementioned databases were imported into Endnote for screening. We first reviewed the titles or abstracts of the articles after removing duplicates, then identified the original studies that were aligned with the present study. We downloaded the full texts, read them thoroughly, and finally screened the studies that were aligned with this systematic review. A standard spreadsheet was created subsequently for later data extraction. The extracted content included title, first author, author's country, publication year, type of study, source of cases, diagnostic criteria for PSD, follow‐up duration, post‐junior high school depression, number of cases, total number of cases, number of PSD cases in the training set, total number of cases in the training set, methods of validation set generation, overfitting, missing value processing, and variable filtering/feature selection, number of post‐junior high school depression cases and number of cases in the validation set, types of models used, and modeling variables.

The above literature screening process was performed independently by Husile and Qinglin Bao, and was cross‐checked after the screening. In the event of any disputes, a third researcher, Temuqile Temuqile would be involved in the decision‐making.

### 3.2 Risk of Bias Assessment

A risk of bias assessment of the included original studies was carried out using PROBAST (Moons et al. 2019). This assessment involved answering a series of questions across four domains: participants, predictors, outcomes, and analysis. These domains reflect overall judgments on risk of bias and applicability. Each domain has a specific number of questions: 2 for participants, 3 for predictor variables, 6 for outcomes, and 9 for statistical analysis. Each question is rated as “yes”, “probably yes”, “no”, “probably no,” or “no information”. If a domain has at least one question answered with “no” or “probably no”, it is considered at high risk. Conversely, if all questions are answered as “yes” or “probably yes”, the domain is considered at low risk. The overall risk of bias will be at low risk if all domains are rated as low risk, and will be at high risk if not less than one domain is rated as high risk.

This assessment was conducted by Husile and Qinglin Bao independently, who cross‐checked the assessments after screening. In the event of any disputes, a third researcher would be involved in the decision‐making.

### 3.3 Outcomes

The primary outcome measures for this meta‐analysis were c‐index, sensitivity, and specificity, which reflect the overall accuracy.

### 3.4 Synthesis Methods

A meta‐analysis on the metric (c‐index) derived from machine learning (ML) models to evaluate their overall accuracy was conducted. In cases where the c‐index was not provided along with the 95% CI and standard error, a study (Debray et al. 2019) by Debray TP, Damen JA, Riley RD, et al. (2019) for estimates of the standard error was referred. Given the variability in ML models and inconsistencies in corresponding parameters, a random‐effects model for meta‐analyses on the c‐index was applied as our primary approach. Additionally, bivariate mixed‐effects models were employed for meta‐analysis of sensitivity and specificity. Sensitivity and specificity were analyzed using the diagnostic chi‐square fourfold table, though this method was not utilized in many original studies. In this case, two methods were applied to construct the table, (1) incorporating sensitivity, specificity, precision, etc. with the number of cases, and (2) extracting sensitivity and specificity based on the optimal Youden's index and calculating based on the number of cases. R4.2.0 (R Development Core Team, Vienna. http://www.R‐project.org) was used for the analysis.

## 4 Results

### 4.1 Study Selection

**FIGURE 1:**
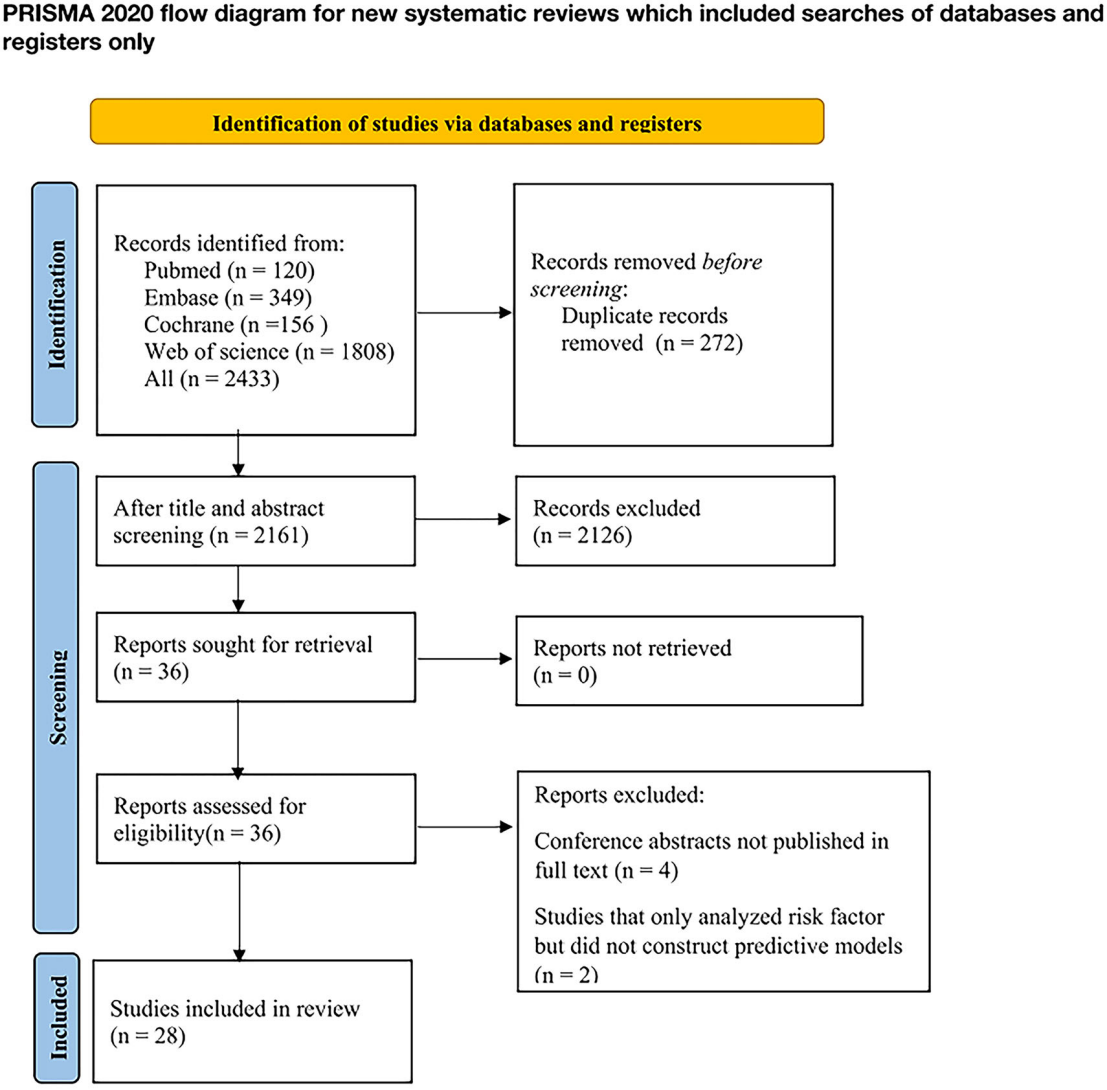
Process of literature screening.

We retrieved 2433 original studies from various databases and extracted 272 duplicate published studies. For the remaining ones, we firstly reviewed the titles or abstracts and excluded 2126 studies that were not relevant to our theme. For the 36 studies that remained, we downloaded their full texts. After reading the full texts, we finally included 28 original studies (Zhu et al. 2022; Zhang et al. 2023; Yao et al. 2023; Wang et al. 2023; Luo et al. 2023; Luo et al. 2023; Gong et al. 2023; Chen et al. 2023; Zhou et al. 2022; Ryu et al. 2022; Qiu et al. 2022; Lan et al. 2022; Ladwig et al. 2022; Yi et al. 2021; Qiu et al. 2021; Che et al. 2021; de Man‐van Ginkel et al. 2013; Hirata et al. 2016; Kootker et al. 2016; Li et al. 2021; Liegey et al. 2021; Liu et al. 2017; Lu et al. 2020; Qiao et al. 2020; Schepers et al. 2009; Unsworth et al. 2019; van de Port et al. 2007; Xu et al. 2018) in the systematic review. Refer to **Figure** **1** for details.

### 4.2 Study Characteristics

Among 28 eligible studies, 19 studies (Zhu et al. 2022; Zhang et al. 2023; Yao et al. 2023; Wang et al. 2023; Luo et al. 2023; Luo et al. 2023; Gong et al. 2023; Chen et al. 2023; Zhou et al. 2022; Qiu et al. 2022; Lan et al. 2022; Yi et al. 2021; Qiu et al. 2021; Li et al. 2021; Liu et al. 2017; Lu et al. 2020; Qiao et al. 2020; Xu et al. 2018) were conducted in China, 4 studies (de Man‐van Ginkel et al. 2013; Kootker et al. 2016; Schepers et al. 2009; van de Port et al. 2007) in New Zealand, 1 study (30928216) in Australia, 1 study (Ladwig et al. 2022) in Germany, 1 study (Ryu et al. 2022) in South Korea, 1 study (Liegey et al. 2021) in France, and 1 study (Hirata et al. 2016) in the United States. The publication years of the abovementioned studies were between 2009 and 2023, primarily in 2020–2023 (n = 20). Among which, 22 were cohort studies (Zhu et al. 2022; Wang et al. 2023; Luo et al. 2023; Luo et al. 2023; Gong et al. 2023; Chen et al. 2023; Zhou et al. 2022; Qiu et al. 2022; Lan et al. 2022; Ladwig et al. 2022; Yi et al. 2021; Qiu et al. 2021; Che et al. 2021; de Man‐van Ginkel et al. 2013; Hirata et al. 2016; Kootker et al. 2016; Li et al. 2021; Liegey et al. 2021; Liu et al. 2017; Lu et al. 2020; Schepers et al. 2009; Xu et al. 2018) and 6 were case‐control studies (Zhang et al. 2023; Yao et al. 2023; Ryu et al. 2022; Qiao et al. 2020; Unsworth et al. 2019; van de Port et al. 2007).

Of the 85,223 subjects included in the studies, 4,241 of them developed post‐stroke depression. The total number of cases in the training set was 71,502, and the number of cases with post‐stroke depression in the training set was 3,682. 6 studies (Luo et al. 2023; Luo et al. 2023; Gong et al. 2023; Chen et al. 2023; Qiu et al. 2022; Yi et al. 2021) carried out independent external validation, mainly by random sampling. The number of cases in the validation set was 16,490, and logistic regression‐based univariate analysis and multivariate analysis were conducted, where the models were still mainly based on logistic regression, but there are a few other types of models as well. The diagnostic criteria in these studies included HAMD ≧ 8 (n = 10) (Zhu et al. 2022; Zhang et al. 2023; Yao et al. 2023; Wang et al. 2023; Luo et al. 2023; Gong et al. 2023; Qiu et al. 2022; Qiu et al. 2021; Che et al. 2021; Kootker et al. 2016), HAMD ≧ 7 (n = 4) (Zhou et al. 2022; Yi et al. 2021; Qiao et al. 2020; Unsworth et al. 2019), etc., and also DMS‐5 (n = 4) (Luo et al. 2023; Ryu et al. 2022; Ladwig et al. 2022; de Man‐van Ginkel et al. 2013), HRSD ≧ 17 (n = 1) (Li et al. 2021), ICD‐10 (n = 1) (Chen et al. 2023), PHQ‐8> 10 (n = 1) (Hirata et al. 2016), as well as blank (n = 7) (Lan et al. 2022; Liegey et al. 2021; Liu et al. 2017; Lu et al. 2020; Schepers et al. 2009; van de Port et al. 2007; Xu et al. 2018), etc. Duration of follow‐up mainly fell in 1 to 6 months, with only one study of 36 months (for prediction of depressive symptoms up to three years post‐stroke).

### 4.3 Risk of Bias in Studies

**FIGURE 2:**
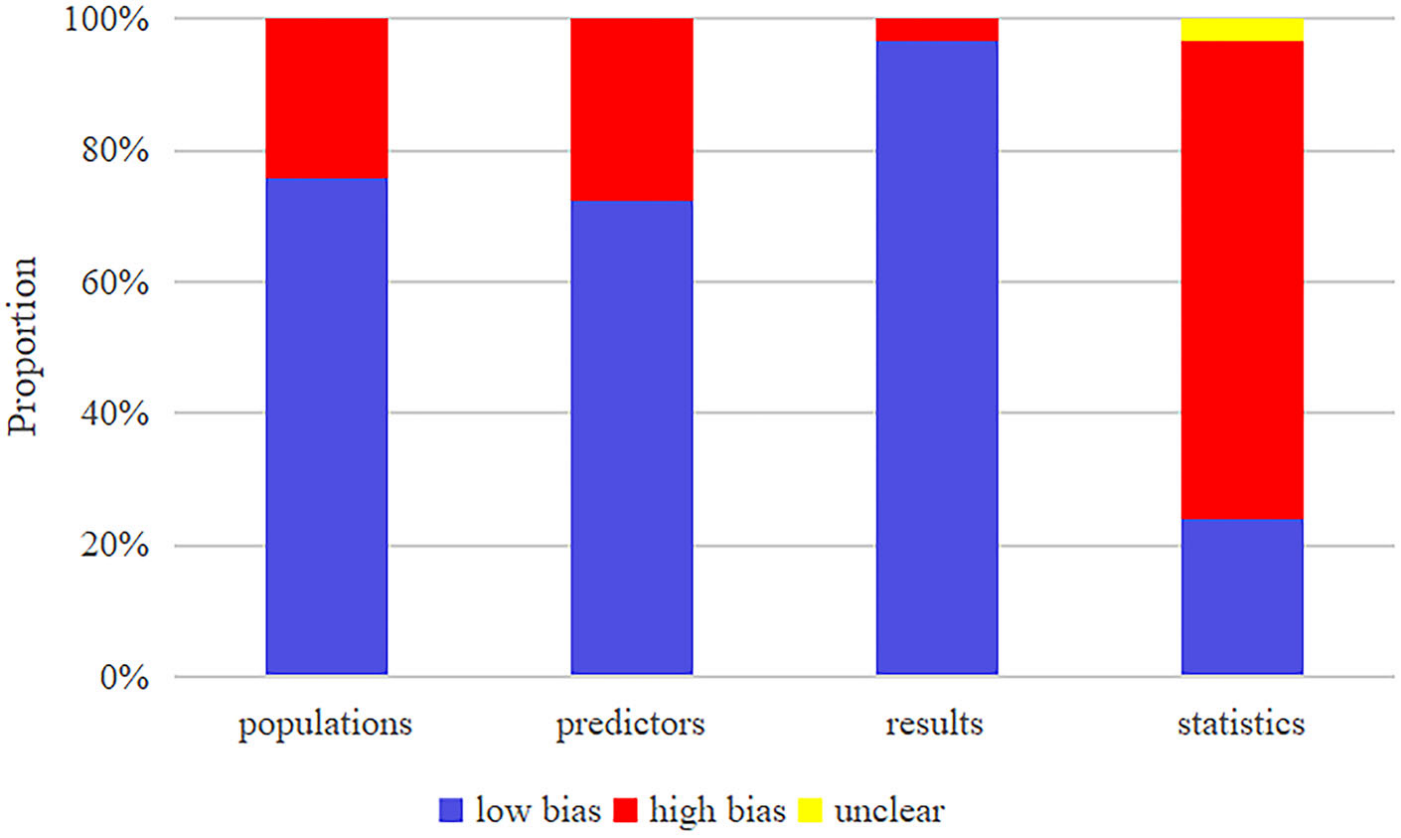
Risk of bias assessment that has incorporated analytical models.

Case‐control studies were judged to be at high risk of bias using PROBAST, and 7 of the models included were from case‐control studies and therefore at high risk of bias. In addition, these case‐control studies present a high risk of bias for the predictor assessment as well. During the diagnosis of post‐stroke depression, those confirmed by ICD‐10 were judged to be at high risk of bias. In terms of statistical analysis, 22 models lacked an independent validation set or had an EPV < 10 and were therefore at high risk of bias (see **Figure** **2**).

## 5 Meta‐Analysis

### 5.1 Synthesized Results

**FIGURE 3:**
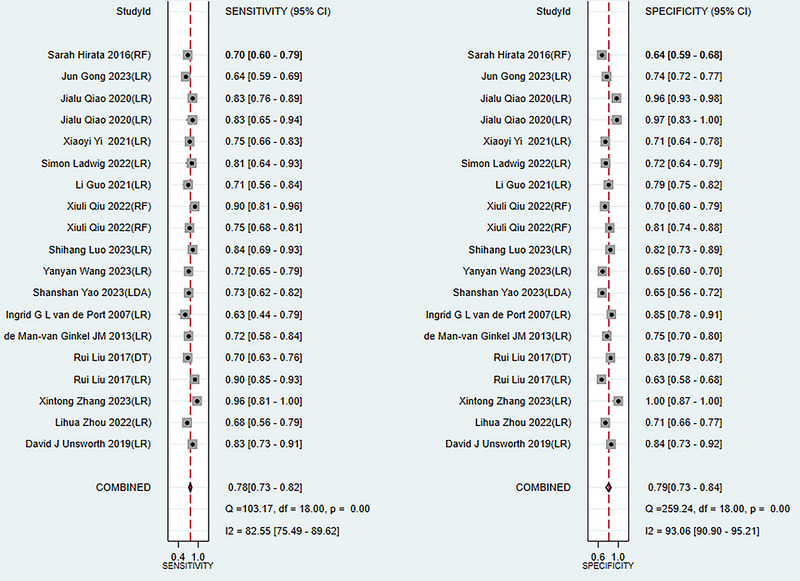
Forest plot of sensitivity and specificity meta‐analysis of models predicting the risk of post‐stroke depression in the training set.

**FIGURE 4:**
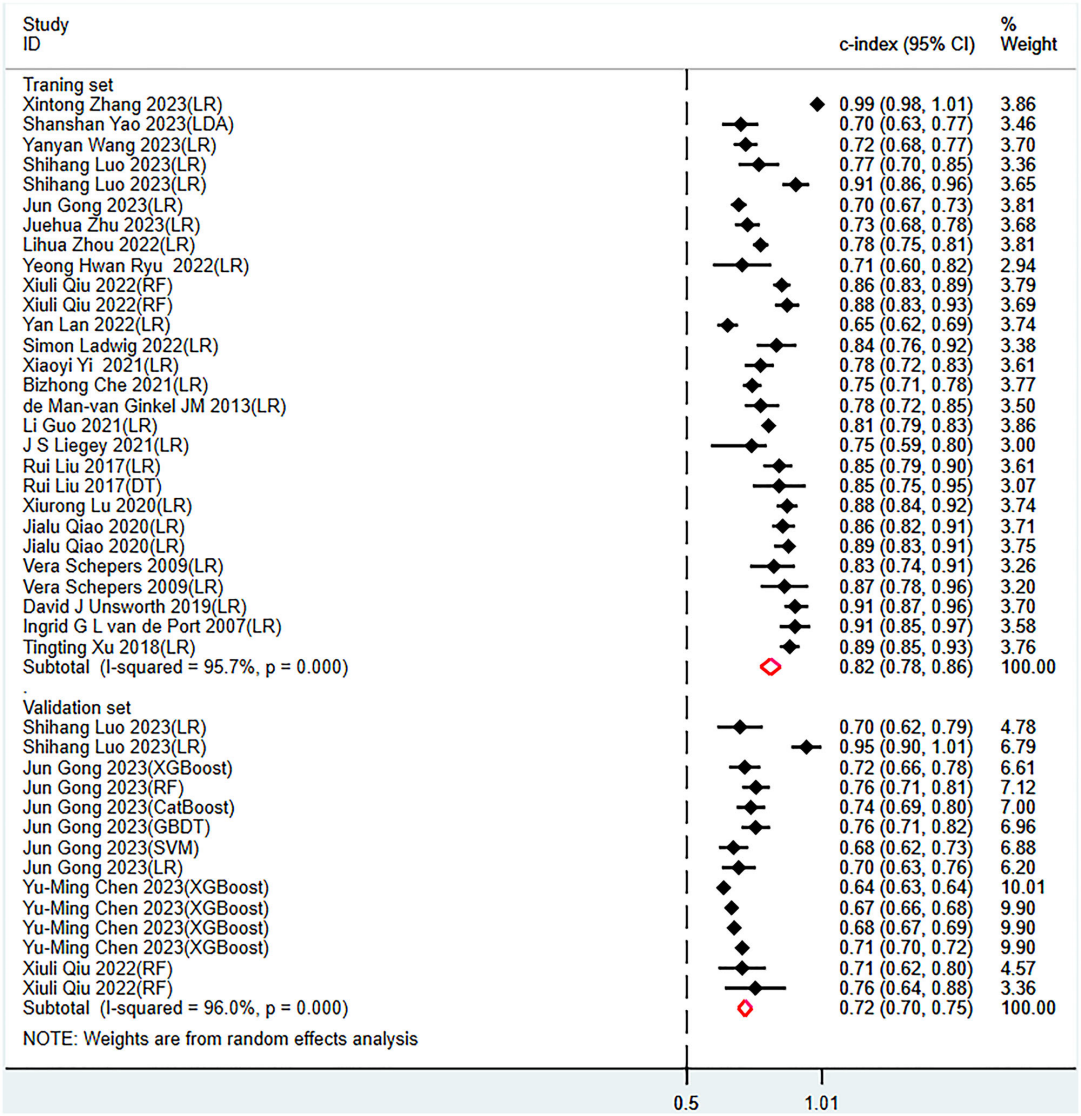
Forest plot of c‐index meta‐analysis of models predicting the risk of post‐stroke. depression in the training and validation sets.

**FIGURE 5:**
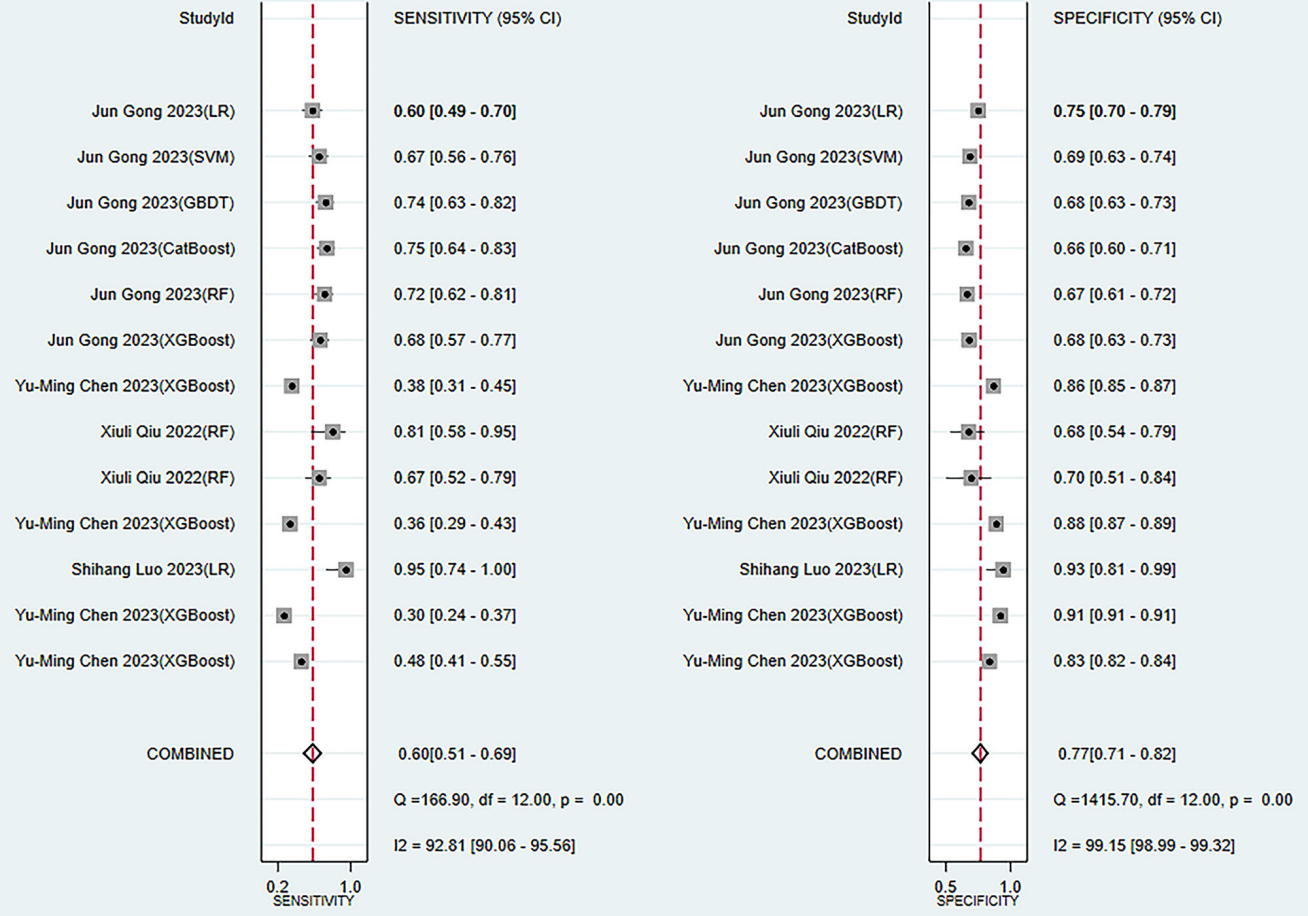
Forest plot of sensitivity and specificity meta‐analysis of models predicting the risk of post‐stroke depression in the validation set.

The meta‐analysis in the training set showed a c‐index of 0.82 (95% CI: 0.78 ‐ 0.86), a sensitivity of 0.78 (95% CI: 0.73 ‐ 0.82), and a specificity of 0.79 (95% CI: 0.73 ‐ 0.84) with the pooled random effects models (see publisher Figure 3). Meta‐analysis in the validation set showed a c‐index of 0.72 (95% CI: 0.70 ‐ 0.75), a sensitivity of 0.60 (95% CI: 0.51 ‐ 0.69), and a specificity of 0.77 (95% CI: 0.71 ‐ 0.82) (see **Figures** 3, 4, 5).

### 5.2 Meta‐Regression and Reporting Biases

**FIGURE 6:**
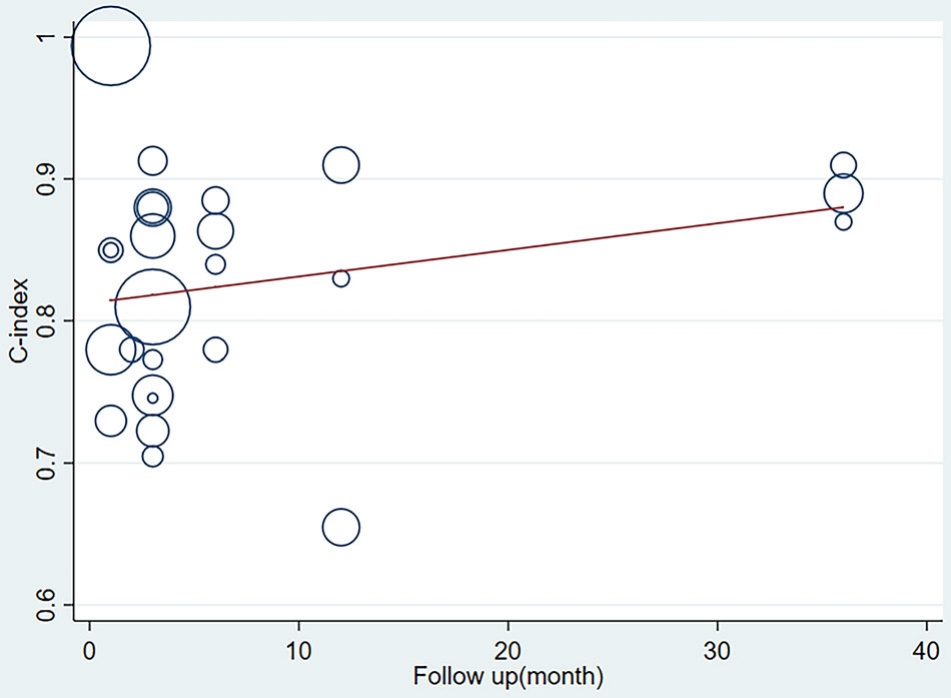
Plot of c‐index and follow‐up duration meta‐regression of models predicting the risk of post‐stroke depression in the training set.

**FIGURE 7:**
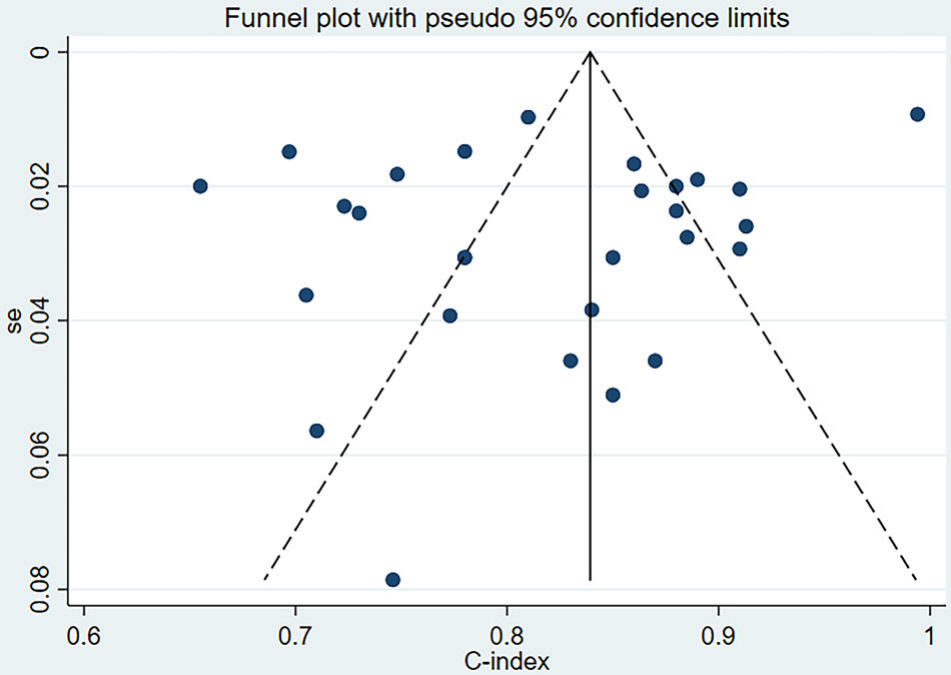
Funnel plot of c‐index meta‐analysis of models predicting the risk of post‐stroke depression in the training set.

Due to the different durations of follow‐up, we need to discuss whether the predictive value and c‐index between the different durations of follow‐up and the built prediction models show significant differences. Therefore, we used meta‐regression, and a meta‐regression indicated that the c‐index was not significantly different at different durations of follow‐up, p = 0.227 (see **Figure** **6**). In the training set, a funnel plot indicated that no publication bias existed in the c‐index. The results of the eager test showed p = 0.017 (see **Figure** **7**).

## 6 Discussion

### 6.1 Summary of the Main Findings

This study investigated the use of machine learning for predicting post‐stroke depression in a training set through prediction models. The pooled random effects model yielded a c‐index of 0.82 (95% CI: 0.78 ‐ 0.86), with a sensitivity of 0.78 (95% CI: 0.73 ‐ 0.82) and a specificity of 0.79 (95% CI: 0.73 ‐ 0.84). In the validation set, the c‐index was 0.72 (95% CI: 0.70 ‐ 0.75), with a sensitivity of 0.60 (95% CI: 0.51 ‐ 0.69) and a specificity of 0.77 (95% CI: 0.71 ‐ 0.82). We found that compared to the training set, the c‐index in the validation set was lower, with a significant difference, suggesting a potential risk of overfitting, despite the overall accuracy being satisfactory.

### 6.2 Comparison With Previous Reviews

Artificial intelligence‐based prediction models demonstrate high accuracy in predicting stroke outcomes (Yang et al. 2023). Machine learning algorithms are capable of integrating various variables, including demographic, clinical, and imaging parameters (Lee et al. 2023). For early clinical prediction of post‐stroke depression, including model scales, several scales are referenced in a Chinese guideline (Zhao et al. 2018): self‐rating scales, such as the Self‐Rating Depression Scale (SDS), Beck Depression Inventory (BDI), Patient Health Questionnaire‐9 (PHQ‐9), and Hospital Anxiety and Depression Scale (HADS). Recent studies have introduced different models, with Yingying Yue et al. identifying the PSDS as a screening tool with acceptable psychometric properties for estimating various subtypes of PSD patients. In addition, DepRes (Hirt et al. 2020) is effective for identifying stroke patients in the early stages who are not at risk of MDD within two months after stroke. However, its limitation lies in the inadequate structure for follow‐up diagnoses in high‐risk patients. Risk factors for PSD include being female, having a history of psychiatric disorders, experiencing major or multiple strokes, injuries to the frontal/anterior regions or basal ganglia, having a stroke within the past year, lacking social support, and facing significant physical disabilities, etc. (Medeiros et al. 2020, Kuang et al. 2022). In particular, histories of mental disorders, physical disabilities, and social support have been identified as independent predictors of post‐stroke depression symptoms (Ladwig et al. 2023). Furthermore, reduced daily living activities may also lead to post‐stroke depression (Li et al. 2023).

Rapidly measurable biomarkers to predict PSD are essential for effectively allocating healthcare resources and enhancing nursing. Recent studies have revealed several predictive biomarkers for PSD. However, these biomarkers lack accessibility in clinical settings (Li et al. 2021). In cases of worsening mood related to PSD, over 80% of mood‐related immune protein levels were found to be elevated, indicating a significantly overactive immune response in chronic stroke patients (Bidoki et al. 2023). A prospective cohort study (Qiu et al. 2022) identified critical biomarkers related to 3‐month PSD: fibrinogen level in males and free T3, magnesium, and BDNF levels in females. Additionally, elevated MHR levels were associated with 3‐month PSD (Li et al. 2022). In addition, studies exploring the relationship between liver function test index and PSD have shown that elevated levels of ALT, TBA, and ALB/GLB increase the likelihood of developing PSD, while rising levels of AST, TBil, and TP decrease the risk (Gong et al. 2023). In acute stroke patients, elevated CRP levels indicate an increased risk of developing PSD (Yang et al. 2022), especially associated with depression symptoms on Day 8 after stroke (Kowalska et al. 2020). Some studies have also found that higher NIHSS scores, elevated homocysteine levels, and multi‐site lesions may serve as independent risk factors for PSD onset in early stages (Zhou et al. 2023). In patients with major depressive disorder (MD), various studies employing structural and functional MRI have revealed significant alterations in the frontal and prefrontal regions, including the orbitofrontal cortex (OFC), dorsolateral prefrontal cortex (dlPFC), anterior cingulate cortex, and subcortical structures. The mood symptoms and prevalence of depression after stroke were notably associated with lesions in the dorsal thalamus, anterior insula, and somatosensory cortex (Krick et al. 2023; Luo et al. 2023). Furthermore, some studies have shown that post‐stroke depressive symptoms are associated with lesions in the right insula, right putamen, inferior frontal gyrus, and right amygdala, and structural disconnection in the right temporal lobe (Klingbeil et al. 2023).

### 6.3 Advantages and Limitations

The present study provides the first comprehensive overview on the predictive accuracy of machine learning for PSD, however, there are some limitations.

Firstly, most of the included studies utilized logistic regression, which raises concerns about potential overfitting. Additionally, only a few studies explored alternative models, preventing us from conducting further subgroup analyses and fully elucidating the results.

Secondly, in previous clinical events, radiomics images have focused mainly on disease diagnosis or a limited number of disease prognoses. With advancements in artificial intelligence and machine learning, radiomics has been integrated into image processing, allowing for both disease diagnosis and prognosis prediction. This shift means that images can now provide insights not only into disease identification but also into disease outcomes, demonstrating promising performance. The modeling variables were mainly derived from clinical characteristics, which have been commonly used in radiomics in previous studies for the diagnosis of stroke lesions, early prediction of outcomes, and long‐term prognostic assessment (Chen et al. 2021). Different structural domains of PSD symptoms are associated with specific structural correlations, (Krick et al. 2023) highlighting the significant prognostic value of radiomics for long‐term outcomes in post‐stroke depression (Oestreich et al. 2022). In the present study, we identified some challenges regarding the real‐world predictive value of prediction models built based on clinical features, suggesting that future research should incorporate radiomics approaches.

Thirdly, there were very few independent validation sets in the studies analyzed regarding PSD, which may limit the interpretation of our validation results.

Finally, we observed that various depression assessment tools could influence outcome predictions. However, due to the limited literature included, the present study could not analyze subgroups based on different depression assessment tools, which represents another limitation.

## 7 Conclusions

A prediction model for PSD based on interpretable clinical characteristics demonstrates a relatively desirable predictive value, but there is still potential for improvement with some risk of overfitting. Therefore, further improvement is needed in future studies.

## Author Contributions

**Husile Husile**: writing–review and editing; writing–original draft; investigation. **Qinglin Bao**: conceptualization. **Sarula Sarula**: resources. **Chu La**: formal analysis. **Wujisiguleng Wujisiguleng**: methodology. **Siqintu Siqintu**: methodology. **Temuqile Temuqile**: supervision.

## Ethics Statement

Ethics approval was not required as this is a meta‐analysis.

## Consent

Written informed consent was not required as this is a meta‐analysis.

## Conflicts of Interest

The authors declare no conflicts of interest.

### Peer Review

The peer review history for this article is available at https://publons.com/publon/10.1002/brb3.70557

## Supporting information




**Table S1**. Literature search strategy Declarations

## Data Availability

The original contributions presented in the study are included in the article, further inquiries can be directed to the corresponding author.
