# Supplementary material for: Accuracy of Machine Learning in Predicting Post‐Stroke Depression: A Systematic Review and Meta‐Analysis
Source: Brain Behav. 2025 May 26;15(5):e70557. doi: 10.1002/brb3.70557 (PMC12105110; doi:10.1002/brb3.70557)
Supplement: Supplementary file 1 — Table S1. Literature search strategy Declarations [file BRB3-15-e70557-s001.docx]

**Table S1.** Literature search strategy

**1.Pubmed**

| Search number | Query | Results |
| --- | --- | --- |
| #1 | "Stroke"[Mesh] | 176898 |
| #2 | (((((((((((((((((Stroke[Title/Abstract]) OR (Strokes[Title/Abstract])) OR (Cerebrovascular Accident[Title/Abstract])) OR (Cerebrovascular Accidents[Title/Abstract])) OR (Cerebrovascular Apoplexy[Title/Abstract])) OR (Brain Vascular Accident[Title/Abstract])) OR (Brain Vascular Accidents[Title/Abstract])) OR (Apoplexy[Title/Abstract])) OR (Brain Infarctions[Title/Abstract])) OR (Brain Infarct[Title/Abstract])) OR (Brain Infarcts[Title/Abstract])) OR (Brain Infarction[Title/Abstract])) OR (Brain Venous Infarction[Title/Abstract])) OR (Brain Venous Infarctions[Title/Abstract])) OR (Venous Brain Infarction[Title/Abstract])) OR (Venous Brain Infarctions[Title/Abstract])) OR (post-stroke[Title/Abstract])) OR (poststroke[Title/Abstract]) | 340270 |
| #3 | ("Stroke"[Mesh]) OR ((((((((((((((((((Stroke[Title/Abstract]) OR (Strokes[Title/Abstract])) OR (Cerebrovascular Accident[Title/Abstract])) OR (Cerebrovascular Accidents[Title/Abstract])) OR (Cerebrovascular Apoplexy[Title/Abstract])) OR (Brain Vascular Accident[Title/Abstract])) OR (Brain Vascular Accidents[Title/Abstract])) OR (Apoplexy[Title/Abstract])) OR (Brain Infarctions[Title/Abstract])) OR (Brain Infarct[Title/Abstract])) OR (Brain Infarcts[Title/Abstract])) OR (Brain Infarction[Title/Abstract])) OR (Brain Venous Infarction[Title/Abstract])) OR (Brain Venous Infarctions[Title/Abstract])) OR (Venous Brain Infarction[Title/Abstract])) OR (Venous Brain Infarctions[Title/Abstract])) OR (post-stroke[Title/Abstract])) OR (poststroke[Title/Abstract])) | 376894 |
| #4 | machine learning[MeSH Terms] | 62779 |
| #5 | (((((((((((((((((((((((((machine learning[Title/Abstract]) OR (Transfer Learning[Title/Abstract])) OR (Deep learning[Title/Abstract])) OR (Ensemble Learning[Title/Abstract])) OR (artificial intelligence[Title/Abstract])) OR (random forest[Title/Abstract])) OR (neural network[Title/Abstract])) OR (neural networks[Title/Abstract])) OR (K-Nearest Neighbor[Title/Abstract])) OR (CNN[Title/Abstract])) OR (Support vector machine[Title/Abstract])) OR (SVM[Title/Abstract])) OR (Gradient Boosting Machine[Title/Abstract])) OR (Nomogram[Title/Abstract])) OR (XGBoost[Title/Abstract])) OR (Adaboost[Title/Abstract])) OR (Decision tree[Title/Abstract])) OR (ResNet-50[Title/Abstract])) OR (ResNet[Title/Abstract])) OR (Naive Bayesian[Title/Abstract])) OR (Multilayer perceptron[Title/Abstract])) OR (Bayesian network[Title/Abstract])) OR (Radiomics[Title/Abstract])) OR (Radiomic[Title/Abstract])) OR (Prediction model[Title/Abstract])) OR (Risk model[Title/Abstract]) | 321324 |
| #6 | (machine learning[MeSH Terms]) OR ((((((((((((((((((((((((((machine learning[Title/Abstract]) OR (Transfer Learning[Title/Abstract])) OR (Deep learning[Title/Abstract])) OR (Ensemble Learning[Title/Abstract])) OR (artificial intelligence[Title/Abstract])) OR (random forest[Title/Abstract])) OR (neural network[Title/Abstract])) OR (neural networks[Title/Abstract])) OR (K-Nearest Neighbor[Title/Abstract])) OR (CNN[Title/Abstract])) OR (Support vector machine[Title/Abstract])) OR (SVM[Title/Abstract])) OR (Gradient Boosting Machine[Title/Abstract])) OR (Nomogram[Title/Abstract])) OR (XGBoost[Title/Abstract])) OR (Adaboost[Title/Abstract])) OR (Decision tree[Title/Abstract])) OR (ResNet-50[Title/Abstract])) OR (ResNet[Title/Abstract])) OR (Naive Bayesian[Title/Abstract])) OR (Multilayer perceptron[Title/Abstract])) OR (Bayesian network[Title/Abstract])) OR (Radiomics[Title/Abstract])) OR (Radiomic[Title/Abstract])) OR (Prediction model[Title/Abstract])) OR (Risk model[Title/Abstract])) | 326477 |
| #7 | Depression[MeSH Terms] | 260632 |
| #8 | ((((Depression[Title/Abstract]) OR (Depressive Symptoms[Title/Abstract])) OR (Depressive Symptom[Title/Abstract])) OR (Emotional Depression[Title/Abstract])) OR (Depressive[Title/Abstract]) | 495363 |
| #9 | (Depression[MeSH Terms]) OR (((((Depression[Title/Abstract]) OR (Depressive Symptoms[Title/Abstract])) OR (Depressive Symptom[Title/Abstract])) OR (Emotional Depression[Title/Abstract])) OR (Depressive[Title/Abstract])) | 543592 |
| #10 | #3 and #6 and #9 | 120 |

**2.Cochrane**

| Search number | Query | Results |
| --- | --- | --- |
| #1 | MeSH descriptor: [Stroke] explode all trees | 15322 |
| #2 | (Stroke):ti,ab,kw OR (Strokes):ti,ab,kw OR (Cerebrovascular Accident):ti,ab,kw OR (Cerebrovascular Accidents):ti,ab,kw OR (Cerebrovascular Apoplexy):ti,ab,kw | 73608 |
| #3 | (Brain Vascular Accident):ti,ab,kw OR (Brain Vascular Accidents):ti,ab,kw OR (Apoplexy):ti,ab,kw OR (Brain Infarctions):ti,ab,kw OR (Brain Infarct):ti,ab,kw | 2161 |
| #4 | (Brain Infarcts):ti,ab,kw OR (Brain Infarction):ti,ab,kw OR (Brain Venous Infarction):ti,ab,kw OR (Brain Venous Infarctions):ti,ab,kw OR (Venous Brain Infarction):ti,ab,kw | 5302 |
| #5 | (Venous Brain Infarctions):ti,ab,kw OR (post-stroke):ti,ab,kw OR (poststroke):ti,ab,kw | 7674 |
| #6 | #1 or #2 or #3 or #4 or #5 | 76192 |
| #7 | MeSH descriptor: [Machine Learning] explode all trees | 938 |
| #8 | (machine learning):ti,ab,kw OR (Transfer Learning):ti,ab,kw OR (Deep learning):ti,ab,kw OR (Ensemble Learning):ti,ab,kw OR (artificial intelligence):ti,ab,kw | 6787 |
| #9 | (random forest):ti,ab,kw OR (neural network):ti,ab,kw OR (neural networks):ti,ab,kw OR (K-Nearest Neighbor):ti,ab,kw OR (CNN):ti,ab,kw | 4294 |
| #10 | (Support vector machine):ti,ab,kw OR (SVM):ti,ab,kw OR (Gradient Boosting Machine):ti,ab,kw OR (Nomogram):ti,ab,kw OR (XGBoost):ti,ab,kw | 2323 |
| #11 | (Adaboost):ti,ab,kw OR (Decision tree):ti,ab,kw OR (ResNet-50):ti,ab,kw OR (ResNet):ti,ab,kw OR (Naive Bayesian):ti,ab,kw | 1079 |
| #12 | (Multilayer perceptron):ti,ab,kw OR (Bayesian network):ti,ab,kw OR (Radiomics):ti,ab,kw OR (Radiomic):ti,ab,kw OR (Prediction model):ti,ab,kw | 6685 |
| #13 | (Risk model):ti,ab,kw | 30866 |
| #14 | #7 or #8 or #9 or #10 or #11 or #12 or #13 | 44118 |
| #15 | MeSH descriptor: [Depression] explode all trees | 18827 |
| #16 | (Depression):ti,ab,kw OR (Depressive Symptoms):ti,ab,kw OR (Depressive Symptom):ti,ab,kw OR (Emotional Depression):ti,ab,kw OR (Depressive):ti,ab,kw | 106662 |
| #17 | #15 or #16 | 106662 |
| #18 | #6 and #14 and #17 | 156 |

**3.Embase**

| Search number | Query | Results |
| --- | --- | --- |
| #1 | 'cerebrovascular accident'/exp | 446482 |
| #2 | stroke:ab,ti OR strokes:ab,ti OR 'cerebrovascular accident':ab,ti OR 'cerebrovascular accidents':ab,ti OR 'cerebrovascular apoplexy':ab,ti OR 'brain vascular accident':ab,ti OR 'brain vascular accidents':ab,ti OR apoplexy:ab,ti OR 'brain infarctions':ab,ti OR 'brain infarct':ab,ti OR 'brain infarcts':ab,ti OR 'brain infarction':ab,ti OR 'brain venous infarction':ab,ti OR 'brain venous infarctions':ab,ti OR 'venous brain infarction':ab,ti OR 'venous brain infarctions':ab,ti OR 'post stroke':ab,ti OR poststroke:ab,ti | 534716 |
| #3 | #1 OR #2 | 648370 |
| #4 | 'machine learning'/exp | 439281 |
| #5 | 'machine learning':ab,ti OR 'transfer learning':ab,ti OR 'deep learning':ab,ti OR 'ensemble learning':ab,ti OR 'artificial intelligence':ab,ti OR 'random forest':ab,ti OR 'neural network':ab,ti OR 'neural networks':ab,ti OR 'k-nearest neighbor':ab,ti OR cnn:ab,ti OR 'support vector machine':ab,ti OR svm:ab,ti OR 'gradient boosting machine':ab,ti OR nomogram:ab,ti OR xgboost:ab,ti OR adaboost:ab,ti OR 'decision tree':ab,ti OR 'resnet 50':ab,ti OR resnet:ab,ti OR 'naive bayesian':ab,ti OR 'multilayer perceptron':ab,ti OR 'bayesian network':ab,ti OR radiomics:ab,ti OR radiomic:ab,ti OR 'prediction model':ab,ti OR 'risk model':ab,ti | 373294 |
| #6 | #4 OR #5 | 594168 |
| #7 | 'depression'/exp | 662434 |
| #8 | depression:ab,ti OR 'depressive symptoms':ab,ti OR 'depressive symptom':ab,ti OR 'emotional depression':ab,ti OR depressive:ab,ti | 686553 |
| #9 | #7 OR #8 | 917766 |
| #10 | #7 AND #8 AND #9 | 349 |

**4.Web of science**

| Search number | Query | Results |
| --- | --- | --- |
| #1 | Stroke (Topic) OR Strokes (Topic) OR Cerebrovascular Accident (Topic) OR Cerebrovascular Accidents (Topic) OR Cerebrovascular Apoplexy (Topic) OR Brain Vascular Accident (Topic) OR Brain Vascular Accidents (Topic) OR Apoplexy (Topic) OR Brain Infarctions (Topic) OR Brain Infarct (Topic) OR Brain Infarcts (Topic) OR Brain Infarction (Topic) OR Brain Venous Infarction (Topic) OR Brain Venous Infarctions (Topic) OR Venous Brain Infarction (Topic) OR Venous Brain Infarctions (Topic) OR post-stroke (Topic) OR poststroke (Topic) | 487804 |
| #2 | machine learning (Topic) OR Transfer Learning (Topic) OR Deep learning (Topic) OR Ensemble Learning (Topic) OR artificial intelligence (Topic) OR random forest (Topic) OR neural network (Topic) OR neural networks (Topic) OR K-Nearest Neighbor (Topic) OR CNN (Topic) OR Support vector machine (Topic) OR SVM (Topic) OR Gradient Boosting Machine (Topic) OR Nomogram (Topic) OR XGBoost (Topic) OR Adaboost (Topic) OR Decision tree (Topic) OR ResNet-50 (Topic) OR ResNet (Topic) OR Naive Bayesian (Topic) OR Multilayer perceptron (Topic) OR Bayesian network (Topic) OR Radiomics (Topic) OR Radiomic (Topic) OR Prediction model (Topic) OR Risk model (Topic) | 3091701 |
| #3 | Depression (Topic) OR Depressive Symptoms (Topic) OR Depressive Symptom (Topic) OR Emotional Depression (Topic) OR Depressive (Topic) | 735437 |
| #4 | #1 and #2 and #3 | 1808 |
